# Feasibility and Acceptability of a Mobile App for Prolonged Grief Disorder Symptoms

**DOI:** 10.32872/cpe.10881

**Published:** 2024-03-28

**Authors:** Anaïs Aeschlimann, Nicolas Gordillo, Taro Ueno, Andreas Maercker, Clare Killikelly

**Affiliations:** 1 Department of Psychology, University of Zurich, Zurich, Switzerland; 2 Department of Informatics, University of Zurich, Zurich, Switzerland; 3 SUSMED Inc., Tokyo, Japan; 4 Department of Psychiatry, University of British Columbia, Vancouver, Canada; Philipps-University of Marburg, Marburg, Germany

**Keywords:** prolonged grief disorder, e-mental health, mobile app, self-monitoring intervention

## Abstract

**Background:**

Mobile apps provide a unique platform for mental health assessment and monitoring. They can provide real time, accessible data on symptoms of mental disorders that may yield rich data for detailed clinical assessment and help individuals gain insight into their current mental state. We developed one of the first apps for tracking symptoms of prolonged grief disorder.

**Method:**

In this pilot feasibility study, we assess the feasibility and acceptability of a new mobile app mGAGE for use once a day for 3 weeks. 27 participants completed mental health assessments at t1 and t2.

**Results:**

Adherence to the app protocol was very high with 100% for the first two weeks of use. A surprising finding was the improvement of grief symptoms at t2. Debriefing interviews revealed general qualitative categories including positive feedback, negative feedback and specific recommendations. Overall, the app was found to be feasible for use for the first two weeks and acceptable for bereaved individuals.

**Conclusions:**

This app could provide valuable data for in depth clinical assessment, may support individuals to gain greater insight into their symptoms and may have a therapeutic effect in terms of improved grief symptoms. Implications for future studies including use in larger intervention studies are discussed.

## Highlights

- Prolonged grief disorder is a new mental health disorder in the ICD-11: This is one of the first mobile apps developed to assess the new ICD-11 PGD.
- The app is found to be easy to use and acceptable by bereaved individuals. This may improve clinical assessment as it provides real time data on the intensity and stability of grief over a 2-week period.
- The act of self-monitoring may have an intervention effect; after two weeks of reporting grief symptoms there was a significant improvement in grief symptoms.

The death of a loved one represents one of the most severe life stressors (Breslau et al., 1998). In a majority of those affected (80-90%), acute grief symptoms dissipate after a certain time, however, approximately 10% experience a prolonged and severe grief reaction (Lundorff et al., 2017). With the introduction of the diagnostic category prolonged grief disorder (PGD) in 2018, the World Health Organization’s International Classification of diseases (ICD-11) allows the diagnosis of disordered grief reactions for the first time. PGD is characterized by two core symptoms relating to longing or yearning for the deceased and persistent preoccupation with the deceased, accessory symptoms of emotional distress and a time and functional impairment criterion. The criteria also take into account varying cultural norms and practices, stating that symptoms must exceed the typical duration and intensity in an individual’s culture and context (Maercker et al., 2013). However, the applicability of the diagnostic criteria, including the time criterion, have not yet been established beyond the Western-European contexts (Stelzer et al., 2020).

If left untreated, individuals affected by PGD are at risk of experiencing a range of further serious health and psychosocial problems including increased rates of cardiovascular problems, high blood pressure, harmful health behaviors, substance abuse, or suicidality (Fujisawa et al., 2010; Kersting et al., 2011; Maercker et al., 2008; Prigerson et al., 2009). Being a relatively recent diagnostic category however, PGD may be difficult for clinicians to differentiate from normal grief (Keeley et al., 2016). Furthermore, PGD does not respond well to interventions that are intended and effective for other bereavement-related mental health problems, e.g., depression, but rather calls for interventions specifically tailored to the precursor to PGD, complicated grief (M. K. Shear et al., 2016). Hence, a correct diagnosis is highly relevant for identifying individuals in need of treatment and providing suitable interventions.

Although recent evidence has shown the effectiveness of interventions specifically tailored to PGD, research still remains limited and further investigation is necessary (Boelen et al., 2006; Bryant et al., 2014; Killikelly & Maercker, 2017; Shear et al., 2005). Another domain of research, which is still lacking, concerns providing the individual with the right support at the right time (Wagner, 2013; Wakefield, 2012). Currently, the long-term trajectories of PGD are poorly understood and further research on the heterogeneity in the fluctuations of symptoms over time is needed (Bonanno & Malgaroli, 2020; Sveen et al., 2018). Some individuals may need immediate psychotherapy support while others may benefit from self-help and monitoring (Johannsen et al., 2019). In addition, recall bias can be problematic for individuals who provide a one-off self-report questionnaire on symptoms. From depression to psychosis there can be variability in the presentation and reporting of symptoms day by day. Digital technology provides the opportunity to more reliably monitor symptoms daily over a longer period of time to ensure a robust assessment and valid measurement (Lenferink et al., 2022). Alongside more accurate diagnosis, symptom tracking and self-monitoring has two uses for interventions. Firstly, daily monitoring alongside psychological and behavioral interventions would allow participants to see the effect of interventions as they unfold day to day. Secondly, the mere act of tracking and monitoring symptoms could provide more insight into symptoms and their severity.

In recent years, digitalization has gained importance in the domain of mental health, bearing the potential to overcome access barriers, as well as expanding the availability and quality of mental health treatment (Chandrashekar, 2018; Torous et al., 2019). Innovative solutions to self-management of mental health problems are especially relevant, given the large treatment gap, meaning that only a small proportion of individuals in need of treatment receive professional help (Kohn et al., 2004). E-mental health interventions delivered via smartphones bring many advantages including immediate support, constant availability, anonymity, low cost, greater access and equity of mental health resources (Olff, 2015).

There has been an increase in the number of available smartphone apps for monitoring and management of mental health symptoms (Wang et al., 2018). This method of ambulant monitoring possesses a number of advantages, which have been demonstrated for various mental health problems (Myin-Germeys et al., 2011; Wichers et al., 2011). This type of data may deliver insights on symptom triggers, relapse signatures, real-time effects of treatment and can provide early detection of change in symptoms (Birchwood et al., 2000; Gleeson et al., 2005; Palmier-Claus, 2011). This has the potential of facilitating earlier interventions, preventing relapse and avoiding hospital admissions, thus also reducing costs (Whelan et al., 2015). Recently ‘My Grief App’ was developed by a Swedish collaborator for bereaved parents after the death of a child (Eklund et al., 2021, 2022). It includes modules focused on psychoeducation and intervention with some brief symptom tracking (one item, grief severity). In a pilot study 13 parents used the app for 4 weeks and it was found to be an acceptable and useful intervention. A follow-up randomized control trial is currently underway to assess the effectiveness in reducing PGD symptoms. In terms of grief monitoring, a recent study confirmed the feasibility and acceptability of grief symptom monitoring using a mobile app and experience sampling methodology (Lenferink et al., 2022). Bereaved individuals responded to PGD symptom questions five times a day for two weeks. Adherence was variable with a high drop out rate of 35-40%. Our current study adds to this new wave of symptom tracking research by developing a mobile app to directly assess ICD-11 PGD symptoms using a validated questionnaire, the International Prolonged Grief Disorder Scale (IPGDS) and using a less intensive monitoring frequency.

## A Mobile Self-Report Tool to Assess PGD: mGAGE

Taking into consideration the current knowledge on the benefits of symptom monitoring and harnessing the potential of digitalization, we designed and developed the *mobile Grief Assessment Guide and E-resource* (mGAGE). mGAGE was designed employing the user-centered design process and two focus groups of bereaved individuals.

The first step in our development process was to design an app for use by bereaved individuals in general, not only those with clinically severe PGD. Focus group participants were recruited from a convenience sample and using the snowball technique and the inclusion criteria included adults who had experienced the death of a loved one at least 6 months ago. The first focus group (*n* = 4) openly explored the need for and qualities of an app for grief, including advantages and disadvantages of such an app. The second focus group (*n* = 4) elaborated on a preliminary design for the app and gathered feedback on the design and specific planned features of the app. Examples of the focus group questions include: what type of information about grief would you like to know? How many questions would you answer? What kind of feedback would be helpful for you?

One important consideration in the further development of this app is the purpose of the app. We have sought to ensure that the user centered design (Schnall et al., 2016) is applied to all stages of the app development. User centered design is defined as an iterative developmental process that incorporates user feedback across different stages of app development. This can be achieved through interviews and focus groups. Related to this, the findings from the current study confirm that it is important to provide participants with the choice of how and when to use the app. Several participants identified the need for personalization of the app. In the next iterative round of development, we will add options to ensure that participants can choose when they complete the app, for how long they would like to use it and how frequently.

Moreover, self-monitoring tools, such as mGAGE, have the potential to promote a more empowered and active role of patients in treatment (Huber et al., 2011). Future research should explore these possibilities in the form of a randomized controlled trial with an active control group and importantly, with a clinical sample of those diagnosed with PGD.

**Figure 1:**
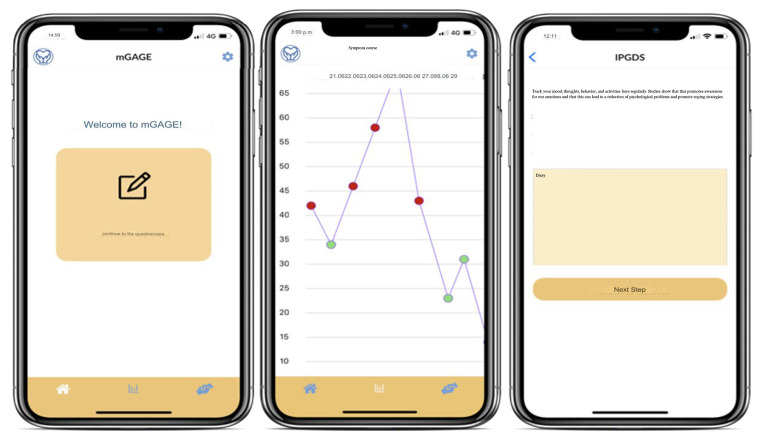
Screenshot of the mGAGE App Welcome Page and Symptom Tracking Screens

In the current version of the mGAGE app it can be delivered via an iOS and Android app, which can be used online on mobile devices. Users create a personalized login ID and are then led to two introduction pages containing information on PGD and the mGAGE app respectively. The app sends users a daily reminder to fill out the integrated questionnaire on PGD symptoms. The completion of the questionnaire takes around 5 minutes. At the end of the questionnaire, users have the option to utilize the diary function, which additionally allows them to record their mood, thoughts, behaviors or actions (see Figure 1). After completion, users receive a feedback concerning the severity of their symptoms, as well as help-seeking recommendations. If the user scores above the cut-off score, indicating that they may suffer from PGD, a direct link to different local services and resources including psychological support appears at the bottom of the page. Furthermore, mGAGE includes a graph function (see Figure 1), which allows users to visualize their assessment history, helping them track the course of their symptoms. Additionally, mGAGE provides users with a support resource page, which lists different professional support resources for bereaved individuals.

The aim of the present study is to (1) evaluate the feasibility of the app in terms of adherence and use of the app as well as (2) the acceptability of the mGAGE app in terms of feedback and evaluation of the app by bereaved individuals. We also explore the variability of IPGDS scores across the 2 weeks and provide insights that may guide the app use to evaluate the effectiveness of PGD interventions.

## Method

### Study Design

We conducted an observational feasibility study with the aim of developing and assessing the feasibility and acceptability of a new mobile grief symptom tracking tool to assist bereaved individuals in self-tracking and monitoring their grief symptoms. The study protocol was approved by the ethics review board of the University of Zurich.

### Participants and Recruitment

Participants were bereaved individuals, who had experienced the loss of a close person. Inclusion criteria specified: 1) fluency in German; 2) age of 18 or older; 3) ability to provide written informed consent; 4) loss of a loved one (family or friend) at least 6 months prior; 5) use of a smartphone. Exclusion criteria were a severe mental health disorder (e.g., Major depression, suicidality, current schizophrenia) or currently being an in-patient.

Participants were remunerated for their time to complete the pre-assessment with 30 Swiss francs (CHF) and the post-assessment with 50 CHF. Furthermore, participants who were Psychology students had the option of being compensated with course credit (4 hours) as an alternative. Additionally, participants had the option of being reimbursed up to 20 CHF for travel expenses related to study participation.

Recruitment took place between August 24^th^ and November 4^th^, 2020. Methods of recruitment included the posting of advertisements and recruitment links on different Facebook groups and mailing lists of the University of Zurich, as well as through WhatsApp groups. Four people who had agreed to participate dropped out before t1. Reasons for drop out included personal reasons and lack of readiness to discuss the death (10 people were interested in the study but did not meet criteria).

### Measures

Participants were assessed twice in-person at the Department of Psychology of the University of Zurich: for the pre-assessment (t1) and the post-assessment (t2). Assessments were conducted by the project manager and a bachelor’s student in Psychology under the supervision of a clinical psychologist. The bachelor’s student was previously trained by the project manager on the study procedures, including the administration of the questionnaires. Additionally, participants were encouraged to provide daily online data regarding their grief symptoms for 3 weeks between t1 and t2. One participant continued to use the app for longer than the required 21 days (up to 27 days). However, this was only 1 participant and beyond the scope of our protocol. The duration of assessment of up to 3 weeks was determined as a limit based on previous experience sampling methods with bereaved individuals who found that adherence to self-monitoring significantly declined after 2 weeks (Lenferink et al., 2022; Mintz et al., 2023).

#### Socio-Demographic Data

Data on socio-demographic information (age, gender, nationality, marital status, education, psychotherapy experience) was collected at t1.

#### Mental Health Outcomes

At t1 and t2, we assessed several mental health outcomes using the measures and descriptions listed here. Grief was assessed using the International ICD-11 Prolonged Grief Disorder Scale, including both the Standard Subscale and the Cultural Supplement, IPGDS Killikelly et al., 2020). The standard subscale consists of 13 items based on the ICD-11 definition of PGD, while the cultural supplement consists of an additional 19 possible items that can be used for treatment planning and cultural acceptability.

Symptoms are rated on a 5-point scale: 1 = almost never (less than once a month), 2 = rarely (monthly), 3 = sometimes (weekly), 4 = often (daily), and 5 = always (several times a day). An impairment item as well as screening items for the length of time since bereavement and the violation of socio-cultural norms was also included. PGD strict criteria requires the fulfillment of the following criteria: one of Items 1 or 2, 1 or more of Items 3-12 and the impairment criteria (Item 13) all rated 4 or above (Lenferink et al., 2021). PGD moderate criteria is the same as the strict criteria except all items are rated 3 or above. Finally, the Maciejewski et al. (2016) criteria includes one of Items 1 or 2, 3-5 of Items 3-12 and no impairment criteria, all rated 4 or higher (Breen & O’Connor, 2007; Maciejewski et al., 2016). Depression: The Patient Health Questionnaire (PHQ) is a self-administered version of the PRIME – MD diagnostic instrument for common mental disorders (Kroenke et al., 2001). The PHQ-9 is the depression module, which scores each of the 9 DSM – IV criteria as “0” (not at all) to “3” (nearly every day). A cutoff score of ≥ 10 has been recommended to indicate moderate to severe depression (Kroenke, Spitzer, & Williams, 2001).

Anxiety: The 7-item Generalized Anxiety Disorder Scale (GAD-7) is a practical self-report anxiety questionnaire that proved valid in primary care according to DSM-IV (Spitzer et al., 2006). Scores for all 7 items range from 0 (Not at all) and 3 (Nearly every day). A cutoff score of 8 or above is recommended to identify possible anxiety disorder.

Post traumatic stress disorder: International Trauma Questionnaire (ITQ) is the ICD-11 based post-traumatic stress disorder (PTSD) measure (Cloitre et al., 2018). The first 9 items of the scale relate to core symptoms of PTSD including re-experiencing, avoidance and sense of current threat and functional impairment. For the purposes of this current study we used only the first 9 items to assess PTSD. For the diagnostic algorithm see Cloitre et al. (2018) Furthermore, we assessed subjective wellbeing (WHO-five wellbeing index, WHO-5, Bech et al., 2003) The total raw score, ranging from 0 to 25, is multiplied by 4: 0 indicates worst well-being and 100 indicates the best well-being. Daily mobile app data regarding grief symptoms was collected using the Standard Subscale from the IPGDS (13 items) which was recently psychometrically validated in terms of reliability and validity (Killikelly et al., 2020). Previous research using similar daily sampling or experience sampling methodology has purported the importance of allowing participants the option to personalize the assessment method and tailor questions based on their current needs or experiences. Here we pilot the use of personalized assessment by including three idiosyncratic, personally relevant items chosen from the cultural supplement alongside the standard scale of 13 items (van Os et al., 2017).

#### Feasibility, Clinical Utility and Acceptability Outcomes

To examine the feasibility of the intervention, we analyzed usage data, specifically (i) percent of participant completing all entries (ii) average entries completed per week. *Acceptability* was assessed during an unstructured exit interview, where participants were asked about their experience using mGAGE and their suggestions for improvement in a potential updated version. Additionally, we employed a questionnaire on acceptability to assess the quality of health-related mobile apps at t2 (mobile app rating scale, MARS, Stoyanov et al., 2015). The MARS evaluates the quality of mobile apps (engagement, functionality, visual aesthetics, information quality and subjective quality subscales) on a scale from 1 (Inadequate) to 5 (Excellent).

### Procedure

Preceding t1, participants were sent an e-mail confirming their appointment for t1, including the study information sheet, the informed consent form, directions to the Department of Psychology and an information sheet about the mGAGE app. Additionally, participants received a reminder e-mail one day before t1.

Assessments were conducted in our offices by the project manager or a Psychology student, who received training on administering the assessment. Participants provided written informed consent. Participants then completed a questionnaire battery about their current mental health status and grief symptoms. Subsequently the participants had the option to select up to three items from the cultural supplement of the IPGDS to be included in their online questionnaire on the mGAGE app. A brief introduction to the app was given where a team member helped participants download the app and create a personal user ID, as well as elaborating all app functionalities and answering any questions participants had (see Supplementary Materials on mobile app information). Participants were instructed to complete the IPGDS within the mGAGE app once a day for three weeks. The IPGDS in this case meant the Standard Subscale of the IPGDS and the potential maximum of 3 additional individual items selected from the cultural supplement of the IPGDS by the participants. Additionally, an information sheet, summarizing all app functionalities was provided. Furthermore, all participants received a list of resources and links for bereaved individuals.

Three weeks after t1, participants were invited to return for the post-assessment t2 and received a reminder e-mail one day before t2. At t2, participants were asked to complete the same questionnaires as for t1 and an additional questionnaire concerning their experience with the mGAGE app. Upon completion, a brief unstructured exit interview was conducted assessing the acceptability of the app. After this, participants received a short debriefing to discuss their experience of participating in the study.

### Analyses

All statistical analyses were conducted in SPSS version 24. Inspection of histograms and the Kolmogorov–Smirnov test statistic (i.e., significance indicates that the distribution of the data significantly differs from a normal distribution) was used to determine whether parametric or non-parametric testing was appropriate. The main outcome measures for grief (IPGDS t1 and t2) were normally distributed while all other measures were normally distributed at one time point. To ensure consistency, the results of the parametric tests (paired samples *t*-tests) were confirmed with non-parametric tests (Mann-Whitney-Wilcoxon test). Feasibility and acceptability variables included percentage of participants completing the daily entry and averaged over the week.

The debriefing interview was analyzed using qualitative thematic analysis. Firstly, the interview data was in vivo transcribed into short relevant sentences and translated into English. Secondly the text was coded and grouped into large categories following iterative categorization (Neale, 2016). Finally, the codes and themes were reviewed by CK and AA and final consensus codes were determined.

## Results

Table 1 presents the demographic characteristics of the participants. Participants were mostly University educated (40.7%) young (average age 25.7) women (88.9%). Time since loss ranged from 6 months to more than 10 years. Almost 30% of participants had previous experience with psychotherapy. Mean scores on the mental health outcome measures are presented in Table 2. Paired sample *t*-tests revealed no significant differences between t1 and t2 on all mental health measures, except for the IPGDS standard scale (13 items) (t1 mean 21.3 vs. t2 mean 18.5, *p* = .002). Table 3 presents the diagnostic algorithm findings. None of the participants met criteria for a strict diagnosis of PGD, while one met criteria for moderate PGD at t1 and two participants at t2. Three participants met criteria for PTSD at t2.

**Table 1:** Sociodemographic, Loss-Related and Symptom Characteristics

Variable	Total sample (*n* = 27)	
	*n*	%
Gender		
Male	3	11.1
Female	24	88.9
Education		
Matura^a^	16	59.3
College/university	11	40.7
Relationship status		
Single^b^	26	96.3
Married	1	3.7
Time since loss		
6 to 12 months ago	8	29.6
1 to 5 years ago	13	48.1
10 to 20 years ago	2	7.4
Other		
Previous psychotherapy for grief^c^	8	29.7
Current psychotherapy^c^	2	7.4

*Note. N* = 27. Participants were on average 25.70 years old (*SD* = standard deviation).

^a^Equivalent to high school education or above in Switzerland. ^b^Is defined here as non-married individuals. ^c^Reflects the number and percentage of participants answering “yes” to this question.

**Table 2:** Questionnaire Data at Pre (T1) Assessment and Post (T2) Assessment

Variable	T1 Pre		T2 Post		Difference test
	*M*	*SD*	*M*	*SD*	Paired samples *t*-test
PGD sum score (13 standard items)	21.3	6.4	18.5	6.3	.002*
PGD cultural supplement	26.5	5.9	26.2	8.7	.779
PHQ9	4.2	2.7	4.4	3.9	.790
GAD7	4.2	3.2	3.8	3.1	.374
ITQ Re	1.3	1.7	1.6	1.9	.387
ITQ Av	1.6	2.2	1.3	2.0	.235
ITQ Th	.6	1.0	.7	1.3	.574
WHO wellbeing scale	15.1	5.2	15.4	4.8	.704
PG13	17.6	5.7	16.8	6.4	.204

*Note. M* = mean; *SD* = standard deviation; PGD = prolonged grief disorder; PHQ9 = patient health questionnaire 9; GAD7 = generalized anxiety disorder 7; ITQ Re = International Trauma Questionnaire – Reexperiencing subscale; ITQ Av = International Trauma Questionnaire – Avoidance subscale; ITQ Th = International Trauma Questionnaire – Threat subscale; WHO = world health organization wellbeing scale; PG13 = prolonged grief 13.

*p < .05. **p < .01. ***p < .001.

**Table 3:** Diagnostic Algorithm Comparison

Diagnostic Test	T1 Pre		T2 Post	
	%	*n*	%	*n*
PGD Strict	0.0		0.0	
PGD Moderate	3.7	1	7.4	2
Maciejewski 2016 criteria	3.7	1	0.0	
PTSD	0.0		11.1	3

*Note.* PGD = prolonged grief disorder; PTSD = posttraumatic stress disorder.

**Figure 2:**
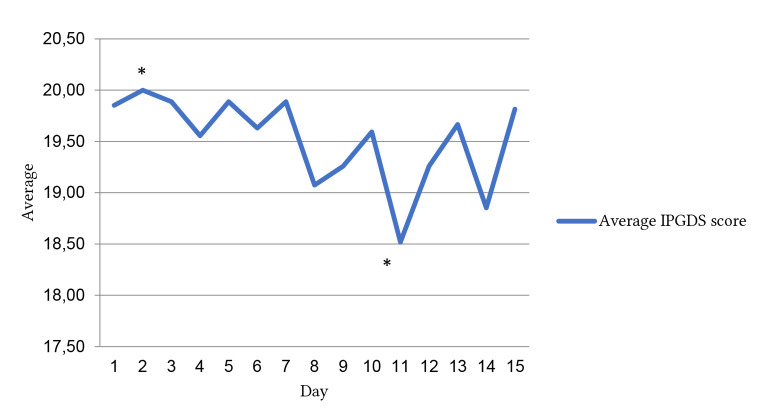
Daily Average IPGDS Score for All Participants *Note.* IPGDS = International Prolonged Grief Disorder Scale. *Statistically significant difference between average daily IPGDS on Day 2 and Day 11; *p* < .05.

**Figure 3:**
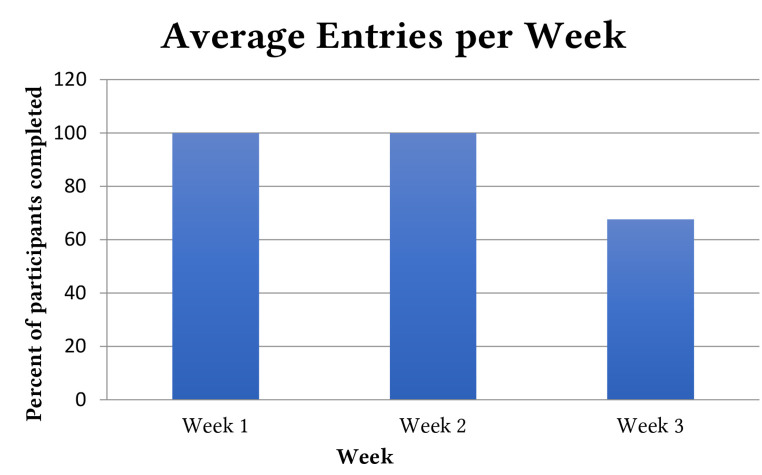
Adherence per Week *Note.* Percent of participants completing the daily questionnaire.

In order to assess the feasibility of mGAGE use, variables related to adherence to the app were examined. Only one participant completed all entries over the total possible 21 days. Up to Day 15 all participants completed the required daily entry. From Day 16 to 21, there was a drop in adherence. As revealed in Figure 3, the average number of entries drops in Week 3 (67.7%) compared to Week 1 and 2 (100%). The average IPGDS score was calculated for Days 1 to 15 (with all data points complete for all participants) to examine the variability in the average score. (See Figures 2 and 3). Paired samples *t*-test compared the highest scored day (Day 2, 20.0) vs. lowest average scored day (Day 11, 18.52) and confirmed a statistically significant difference (*p* = .049).

### Acceptability of mGAGE

Overall mean scores on the MARS questionnaire ranged from 3 to 5 indicating average to very good acceptability for the app (across all questions mean score of 4). The lowest scored question was ‘would you pay for this app’ (average 2). The highest scored questions were ease of use, navigation and gestural designs (all scored average of 5). For the question ‘overall star rating’ (Question 11) participants’ mean score was 4/5.

Thematic analysis was conducted on the debriefing interviews. Overall, three broad categories of responses were identified, each with related themes (see Table 4 for exemplar quotes).

**Table 4:** Summary of Qualitative Feedback and Example Quotes

Themes	Example quotes
Positive Feedback	
Useful links	especially the links to get help were helpful,
Self-monitoring and reflection	But she liked the app, it helped her to reflect and process her grieving thoughts.
Ease of Use	easy to use, also good for older people, fulfilled its purpose,
Good design	the design was very simple but good;
Negative Feedback	
Effect on symptoms	He said even though he thought, that he had already processed his grief symptoms, the app made him a bit sad in the beginning
Notifications and technical issues	the reminders didn't work on a daily basis. Some days the app reminded her, some days not
Repetitive	After two weeks he thought, that the questions were getting boring and annoying, cause they were always the same
Design issues	but the design wasn't that appealing
Specific recommendations	
Personalization and timing	the participant would have liked the app to react to the questions and ask more specific questions
Variation	she would have preferred the questions to be in a different order each time, cause it was a bit repetitive
Other functions	tips like breathing exercises for when score is red (instead of just contact numbers)
IPGDS changes	the IPGDS should have more gradations or possibly a slider bar for more sensitivity
Chart improvement	it would be nice to have a summary of the scores if the app should be used for a longer period of time (e.g. a smaller graph)
Other	
	Even though she liked the app, she wouldn't pay for it.
	Her grief symptoms aren't as strong anymore as they used to be, that's why she didn't find the questions disturbing.

*Note.* IPGDS = International Prolonged Grief Disorder Scale.

 The first category was ‘positive feedback’ with related themes including *useful links, self-monitoring and reflection, ease of use, and good design*. The second category was ‘negative feedback’ with related themes including *effect on symptoms, notifications and technical issues, repetitive, and design issues.* The third category ‘specific recommendations’ included related themes such as *personalization and timing, variation, other functions, IPGDS changes, chart improvement.*

## Discussion

### Main Findings

This pilot study provides preliminary support for the feasibility and acceptability of the mGAGE app for use by bereaved individuals. Feasibility, assessed through adherence to the app, was extremely high for the first two weeks of use (100%). Acceptability, assessed by high ratings on the mobile app rating scale (MARS) questionnaire (mean score of 4/5), was also confirmed. Qualitative feedback from debriefing interviews revealed several themes supporting the acceptability of the app (useful links, self-monitoring and reflection, ease of use, and good design). However, participants also identified possible negative effects on mood as well as specific recommendations for improving the app. The final aim of this study was to explore variability in grief symptoms over two weeks. Here we confirmed high variability (a statistically significant difference between highest and lowest mean scores) over the two weeks. This has important implications for better understanding variation and intensity of grief scores in real time as outlined below. Additionally, the process of self-monitoring may have a therapeutic effect. At t2 participants had significantly lower scores on the International Prolonged Grief Disorder Scale (IPGDS). This significant reduction in symptoms may indicate that online self-monitoring is a beneficial intervention and could be used to supplement face to face therapy, which is in accordance with results from previous studies (e.g. Bakker & Rickard, 2018; Kauer et al., 2012). However, this result should be interpreted with caution as no control group was included in this study. The finding that one participant met moderate diagnostic criteria for PGD and two participants met criteria for PTSD at T2 although not at T1 attests to the need for a control group to unpick the effect of PGD symptom monitoring compared to other possible confounding or moderating effects on T2 outcomes.

Previous research has confirmed that adherence to e-mental health apps wavers. Response rates of around 60-80% have been found in previous e-mental health studies of depression (70%; Putnam & McSweeney, 2008), substance misuse (88.8%; Phillips et al., 2014), and trauma (67.5%; Dewey et al., 2015). Evidently the current study found exceptionally high adherence; 100% of responses completed by all participants. However, after two weeks adherence declines substantially (Week 3 average response 67.7%). This is also found in other areas of e-mental health. After an initial burst of interest participants typically decrease their use of health apps after two weeks (Dorsey et al., 2017). Reasons for decline in use include poor user centered design, lack of incentive and decreased internal motivation (Torous et al., 2019). Importantly, it should be noted that many participants mentioned technical problems as a reason for decline in use. The current study developed the mGAGE app following a user centered iterative design and we plan to build on the current findings to redesign elements of the app to improve acceptability. Mohr et al. (2011) developed a model of ‘supportive accountability’ which seeks to improve adherence to e-mental health interventions by adding person-to-person contact throughout the intervention. They argue that participants are more likely to continue the intervention if they experience contact with a ‘coach’ who is supportive, trustworthy and kind. To improve adherence to the current app we would consider adding contact with a weekly coach to check-in and provide support and encouragement. However, based on the current findings we may consider limiting the required use of the app to two weeks. After two weeks participants have provided daily real-time data on their grief symptoms. This may be enough data to capture a comprehensive clinical picture for further assessment and treatment by a clinician. Additionally, this may provide the individual with more insight into their current symptoms.

One interesting finding of the current study was the possible therapeutic effect of self-monitoring. Previous literature has revealed that the repeated act of completing an outcome measure may be an intervention in itself (Amble et al., 2015). This study found that participants had decreased grief scores at t2. Previous studies have also found that daily self-monitoring may improve mental health symptoms. For example, after 30 days of mood monitoring with a new mobile app, MoodPrism, participants experienced a significant decrease in symptoms of depression and anxiety, and this was directly related to app use (Bakker & Rickard, 2018). The act of daily self-reflection and improved insight into symptoms is the goal of several cognitive-behavioral therapy interventions for grief and other disorders (Boelen et al., 2011; Kavanagh, 1990; Spuij et al., 2015). These are found to be predictors of positive affect, cognitive reappraisal as well as emotional self-awareness (Kauer et al., 2012; O’Toole et al., 2014). Emotional self-awareness is thought to be a key factor that may improve self-regulation and wellbeing (Barrett & Gross, 2001).

Overall participants rated the app as highly acceptable. However, they identified some important areas for improvement. Several participants identified that daily self-monitoring may not have a positive effect on mood, but instead remind bereaved individuals of their sadness and grief. One participant identified that she thought she experienced more negative symptoms after using the app daily. Another worried that it could be difficult for people to be reminded of their loss everyday (see Table 4). This is an important consideration and will be an important topic for the next phase of research, particularly when conducting research with a clinical sample (Wykes & Brown, 2016). As mentioned above, we would consider adding the model of supportive accountability, not only to improve intrinsic motivation to complete the app but to offer participants support, as well as for risk assessment.

### Clinical Implications and Future Research

Overall, the findings of this pilot study confirm that the mGAGE app may have an important use in future randomized controlled trials and grief interventions. Firstly, it can deliver real time daily data on symptom variability and intensity, which provides researchers with a tool to accurately investigate the heterogeneity in the fluctuations of PGD symptoms over time in future research (Bonanno & Malgaroli, 2020; Sveen et al., 2018). This may also be used to inform clinical assessments and treatment options by providing more depth and richer information than a singular self-report assessment. Secondly, daily monitoring can be used to track the effectiveness of grief interventions. As change in symptom severity may vary and effects may waver, mGAGE could be used as an addition to standard, face-to-face therapy and help to track progress and effectiveness, as suggested by Torous and Roux (2017). It could also be used in conjunction with existing online grief interventions (Wagner et al., 2006). Additionally, mGAGE has the potential to aid clinicians in identifying individuals with PGD. By facilitating early identification of cases and accelerating access to appropriate treatment, it could also prevent hospitalization or relapses (Whelan et al., 2015). Thirdly, the act of self-monitoring may be a useful intervention by increasing emotional self-awareness. Developing insight into the severity and variability of symptoms may be an effective therapeutic tool (Bakker & Rickard, 2018).

### Limitations

This study was limited in the following ways. The sample size was small and homogeneous. It was a largely female sample of the same age group and education level. We did not include individuals from a clinical sample. This is the next required step to ensure acceptability for PGD diagnosis. There were also several technical issues with the app (such as server unavailable, only worked with internet connection) that may have prevented optimal data collection. In terms of the qualitative debriefing interviews, there may have been a social desirability effect as the participants were not blinded to the interviewer. The finding that two participants met criteria for PTSD or moderate PGD at T2 needs further investigation. Currently no participants at t1 or t2 met ‘strict’ criteria for PGD is reassuring as our intention was not to investigate a clinical sample in this pilot study. The diagnostic algorithm for PGD is currently under debate with no clear consensus on whether the ‘strict’ ‘moderate’ or another algorithm for ICD-11 PGD may yield the most reliable and valid diagnosis (Boelen & Lenferink, 2020). In the present study if a participant met criteria for moderate PGD, currently this does not necessarily indicate disorder. However, it may mean elevated symptoms. This should be monitored and follow up with a case control analysis or an RCT including a control group. Another significant limitation in the sample that requires follow up is the heterogeneity in the time criteria. Time since death is a significant predictor of grief symptom severity and although all included participants experienced a death more than 6 months ago, there was still large variability in the duration since loss. The impact of time since loss on daily sampling and grief symptom variability should be assessed in future studies.

## Supplementary Materials

The Supplementary Materials contain the following item (for access, see Aeschlimann et al., 2024):

- Information pamphlet in German and English describing the functions and features of the mGAGE app for prolonged grief disorder.

AeschlimannA.
GordilloN.
UenoT.
MaerckerA.
KillikellyC.
 (2024). Supplementary materials to "Feasibility and acceptability of a mobile app for prolonged grief disorder symptoms"
[Information pamphlet]. PsychOpen. 10.23668/psycharchives.14238
PMC1130391339119221
